# Can *Paulownia* Siebold & Zucc. Become an Invasive Species via Its Seeds?

**DOI:** 10.3390/plants15070989

**Published:** 2026-03-24

**Authors:** Jiří Kadlec, Kateřina Novosadová, Kateřina Macháčková, Petr Sýkora, Radek Pokorný

**Affiliations:** Department of Silviculture, Faculty of Forestry and Wood Technology, Mendel University in Brno, Zemědělská 3, 613 00 Brno, Czech Republic; katerina.novosadova@mendelu.cz (K.N.); katerina.machackova@mendelu.cz (K.M.); xsykora@node.mendelu.cz (P.S.); radek.pokorny@mendelu.cz (R.P.)

**Keywords:** germination rate, germination energy, hybrids, *P. tomentosa*, *P. elongata*, *P. fortunei*

## Abstract

*Paulownia* plantations are established in large numbers worldwide for their high production of quality wood. *Paulownia tomentosa* is considered an invasive plant in many countries; however, other species, and mainly their hybrids that grow in plantations, are classified neither as invasive nor non-invasive plants, although the risk of spontaneous spreading of its seeds can be very great. In April and September 2022, we conducted a germination experiment where we used three species and six hybrids of *Paulownia*. The germination rates of all selected species and hybrids were very high, especially if the seeds were left at a temperature of +4 °C—almost 90% (April) and around 60% (September). When the seeds were exposed to below-zero temperatures (i.e., −15 °C), the germination rates were still high and, moreover, those of Hybrids were higher than those of Species. Therefore, all species of *Paulownia*, and mainly the hybrids, have the potential to be invasive.

## 1. Introduction

The taxonomy of individual plants of the *Paulownia* genus (Siebold & Zucc.) is very complicated. This is because there are several traits according to which they are classified under individual families. In 1781, ThunBerg classified *Bignonia tomentosa* Thunb. and its entire genus under the family *Bignoniaceae* Juss. In 1835, Zuccarini and Siebold transferred this genus under the family *Scrophulariaceae* Juss, and gradually the genus grew to as many as 23 species. In 1959, more detailed research was conducted, and only six species remained. To this date, however, there have been discussions regarding the similarity and classification of individual species. Therefore, the *Paulownia* genus is either classified under the *Paulowniaceae* Nakai [1] or assigned to the *Scrophulariaceae* [2,3] or *Bignoniaceae* [2,4] family. Moreover, the *Paulownia* genus is divided into three types, according to the degree of cultivation [5,6,7,8], namely:

- **Wild**, which are unsuitable for plantations because the species do not have good growth characteristics, for example, *Paulownia albiphloea* Z.H. Zhu sp.nov, *P. catalpifolia* T. Gong ex D.Y. Hong, or *P. kawakamii* T.Itô (syn.: *P. rehderiana*, *P. thyrsoidea*, *P. viscosa*) [2,6,9];
- **Semi-wild**, species (hereinafter referred to as Species) that have better growth characteristics, greater resistance to disease than wild types, and, moreover, can be planted in smaller plantations, for example, *Paulownia fortunei* (Seem) Hemsl, *P. elongata* S.Y. Hu, and *P. tomentosa* (Thunb.) Steud. [2,6,9];
- **Art**, which are kinds of *Paulownia* (hereinafter referred to as Hybrid) with a great yield of quality wood, have been artificially selected and planted since the 1980s [6,7,8]. Currently, there are dozens of hybrids that have different growth characteristics and have been cultivated for various regions with different climates.

*Paulownia* is originally from China, which means that it had been introduced to Europe and America [2]. It starts to produce seeds at the age of 4–10 [10,11,12], and produces up to 20 million seeds per year [13,14]. These seeds survive 2–3 years on the soil surface and up to 15 years in the soil [10,15,16,17], and they have a high emergence rate [10,18]. They are transported by wind and water over considerable distances [19,20]. New plants commonly occur almost 4 km from the parent tree [18], and have been located even at a distance of 10 km [10].

Non-native plants that had been introduced up to 1492 are called “archaeophytes”, later “neophytes” [21]. If these non-native plants have an undesirable impact on native plants, they are called “invasive” [22]. There is no official definition of “invasive tree species”. There are many versions stated by individual authors. For example, invasive species are the following:

- Naturalized plants that produce offspring (often in very large numbers) at considerable distances (even more than 100 m in less than 50 years for species spreading by seeds or more than 6 m in 3 years for species spreading by vegetative reproduction) [23].
- Plants capable of surviving, reproducing, and spreading across landscape, sometimes at alarming rates [24].
- “Alien” species that settle down in natural or semi-natural ecosystems or habitats. They are a change agent that threatens native biological diversity [25].
- “Alien” species that must successfully out-compete native organisms for food and habitat, spread through their new environment, increase their population, and harm ecosystems [26].

The environmental impact of invasive plants is assessed using Environmental Impact Classification for Alien Taxa (EICAT) [27]. It is important to distinguish non-native plants that are

- Cultivated in forests, for example, *Pinus strobus* L. (major EICAT score) [28]; *Robinia pseudoacacia* L. (massive EICAT score) [29]; or *Quercus rubra* L. (major EICAT score) [30].
- Not legally permitted to be grown in forests, for example, *Ailanthus altissima* (Mill.) Swingle (major EICAT score) [31].
- Neither legally permitted nor prohibited (for example, *Paulownia* ssp.).

The designation of *P. tomentosa* as an invasive or casual species is a subject of discussion. Numerous authors [32,33,34,35,36] have conducted ecological assessments of *Paulownia* in European and North American regions, from which there are no definitive statements about its invasiveness. It is not included in the updated list of invasive alien species of the European Union (European Commission) [37]. However, a number of studies [15,38,39,40,41,42] state that *Paulownia* is spreading across Europe and the USA. The European and Mediterranean Plant Protection Organization [43] included *P. tomentosa* on the “Alert List” of geographically non-native organisms. In the Czech Republic (CR), *P. tomentosa* acquired the status of an alien species requiring constant monitoring [42], and Pergl et al. [44] classified it into the “Watch List” (i.e., a list of the plants that have a potentially large impact on the environment). It has been found in other European countries with similar natural conditions, but it does not occur in the wild in the CR. It was legally considered invasive in Austria [32] and in the USA [45]. Less is published about *P. elongata*, and all other species and hybrids of *Paulownia* are (usually) omitted. According to some opinions, *P. elongata* is not considered an invasive species, but is accepted reluctantly [38,42]. However, Huber et al. [21] show that, due to global climate change, it can become invasive. The invasiveness of *Paulownia* hybrids is not taken into consideration at all, although there is a risk of their spread from the plantations, which are currently being established in large numbers.

Therefore, we set out to test and confirm two assumptions:

(I) There is a difference between individual species/hybrids in seed germination.
(II) There is the danger that more species and hybrids could be invasive (besides *P. tomentosa*).

## 2. Results

### 2.1. Germination Rate (GR)

**Figure 1:**
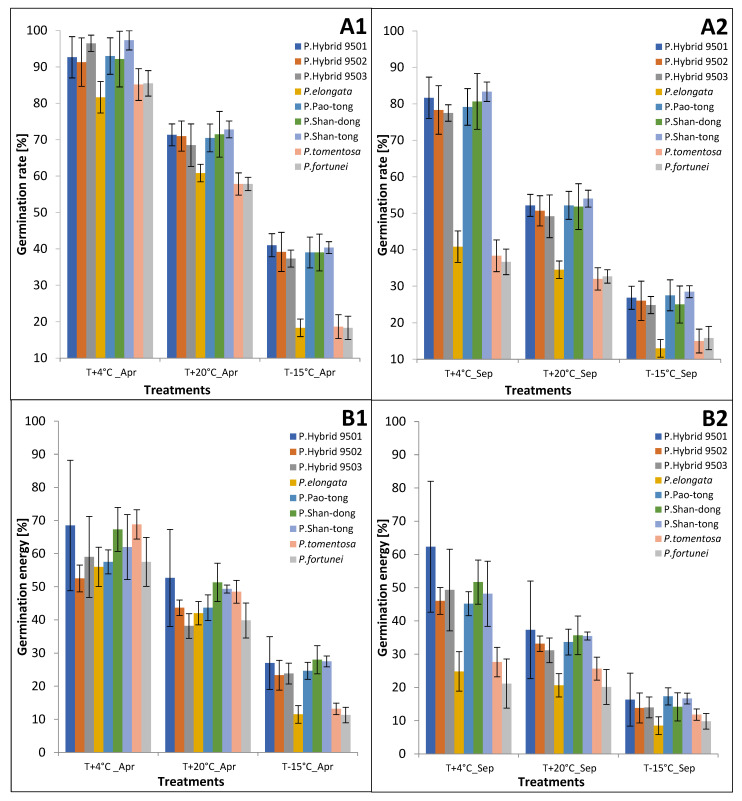
Germination rates (**A1**,**A2**) and germination energies (**B1**,**B2**) of *Paulownia* species and hybrids. 1—in April; 2—in September; T+4 °C—treatment at +4 °C temperature; T+20 °C—treatment at +20 °C temperature; T−15 °C—treatment at +4 °C and −15 °C temperatures; Apr—in April; Sep—in September. Whiskers denote standard deviations.

The treatments of seeds that germinated in April always had higher GRs than those in September (Temperature+4 °C: *p* = 0.0001–0.0072; Temperature+20 °C: *p* = 0.0001–0.0103; Temperature−15 °C: *p* = 0.0001–0.0047; Figure 1). The highest GRs always appeared in the Temperature+4 °C treatment (from 82 to 97% in April and from 37 to 83% in September), and there were differences among all Species and Hybrids, where Species had lower GRs (April: *p* = 0.0071–0.0349; September: *p* = 0.0138–0.0418). The second highest GRs were recorded in the Temperature+20 °C treatment (from 58 to 73% in April and from 32 to 54% in September). There were differences among all Species and Hybrids, where Species had lower GRs (April: *p* = 0.0287–0.0473; September: *p* = 0.0249–0.0464). In the Temperature−15 °C treatment, the GRs were the lowest of all treatments. The GRs of all Hybrids were similar (around 38% in April and 26% in September, without statistically significant differences among them) and also higher than those of Species (around 18% in April and around 14% in September, without statistically significant differences among them). The statistically significant differences among Hybrids and Species were between 0.0089 and 0.0254.

### 2.2. Germination Energy (GE)

In both April and September, GE was always highest in the Temperature+4 °C treatment and the second highest in the Temperature+20 °C treatment (Figure 1). There were statistically significant differences between the corresponding GEs of *P*. *elongata*, P. Pao-tong, P. Shan-tong, *P. tomentosa*, and *P. fortunei* in these two treatments (*p* values were from 0.0479 to 0.0001 in April and from 0.0412 to 0.0001 in September). The lowest GEs occurred in the Temperature−15 °C treatment. There were statistically significant differences between the corresponding GEs of all Species and Hybrids in the Temperature+4 °C and Temperature−15 °C treatments (*p* values being 0.0001) and between the corresponding GEs of all Species and Hybrids (*p* values being 0.0001) in the Temperature+20 °C and Temperature−15 °C treatments.

In the Temperature+4 °C treatment, the GE values varied in the range of 53–69% in April and 21–62% in September. In April, the highest values were recorded on the seeds of *P. tomentosa* and P. Shan-dong. Paulownia Hybrid 9502, *P. elongata*, P. Pao-tong, P. Shan-tong, and *P. fortunei* had the second highest values (*p* values of the individuals in this group and those in the highest one were in the range of 0.0001–0.411). The smallest values were found on the seeds of P. Hybrid 9503 (*p* values of P. Hybrid 9503 and the individuals in the other groups (i.e., the highest group and the second highest group) were in the range of 0.0001–0.0008). In September, the highest value was recorded on the seeds of P. Hybrid 9501. Paulownia Hybrid 9502, P. Hybrid 9503, P. Pao-tong, P. Shan-tong, and P. Shan-dong had the second highest values (*p* values of the individuals in this group and those in the highest one were in the range of 0.0157–0.0397). The smallest values were found at seeds of the others (*p* values of the individuals in this group, and those in the highest one were in the range of 0.0001–0.0032).

In the Temperature+20 °C treatment, the GE values varied in the range of 38–53% in April and 20–37% in September. In April, the highest values were recorded on the seeds of P. Hybrid 9501, P. Shan-tong, P. Shan-dong, and *P. tomentosa*. The other Hybrids and Species had statistically smaller values than those in the first group (*p* values of the individuals in the first group and those in the second group were in the range of 0.0001–0.0473). In September, the highest values were recorded on the seeds of all Hybrids, and all Species had statistically smaller values than the Hybrids (*p* values of Hybrids and Species were in the range of 0.0315–0.0482).

In the Temperature−15 °C treatment, the GE values varied in the range of 11–28% in April and 9–17% in September. In April, the highest values were recorded on the seeds of all the Hybrids, and all Species had statistically smaller values than the Hybrids (*p* values of Hybrids and Species were in the range of 0.0137–0.0439). In September, the highest values were recorded on the seeds of all the Hybrids, and all Species had statistically smaller values than the Hybrids (*p* values of Hybrids and Species were in the range of 0.0234–0.0401).

### 2.3. The Dynamic of Germination

**Figure 2:**
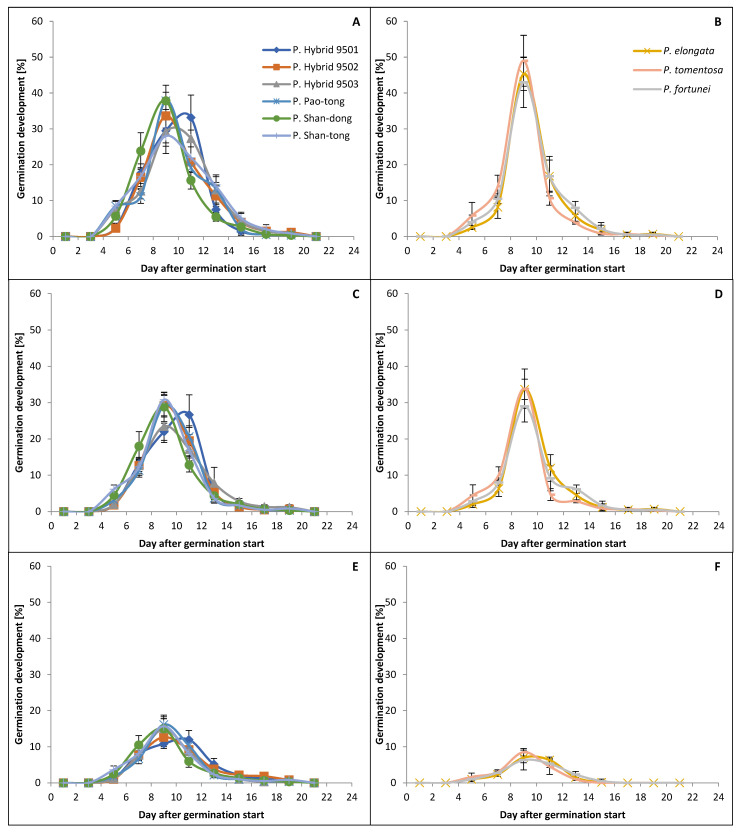
Dynamic of germination of *Paulownia* in April. (**A**,**B**)—the Temperature+4 °C treatment; (**C**,**D**)—the Temperature+20 °C treatment; (**E**,**F**)—the Temperature−15 °C treatment. Whiskers denote standard deviations.

**Figure 3:**
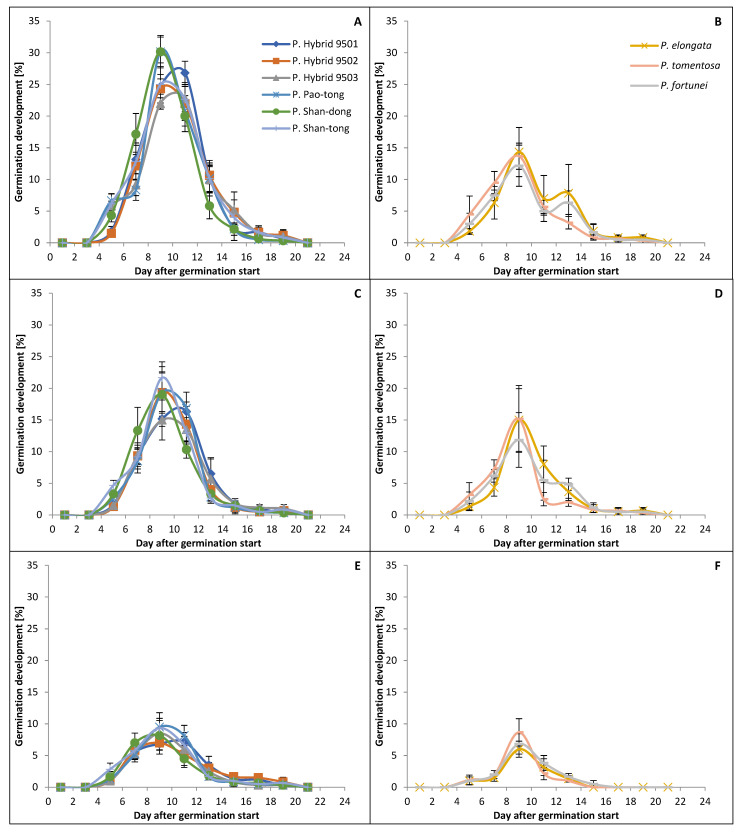
Dynamic of germination of *Paulownia* in September. (**A**,**B**)—the Temperature+4 °C treatment; (**C**,**D**)—the Temperature+20 °C treatment; (**E**,**F**)—the Temperature−15 °C treatment. Whiskers denote standard deviations.

The germination (both in April and September) began each time no later than the fourth day after the start of the experiment and ended no later than the eighteenth day (Figure 2 and Figure 3). The germination peaks of Hybrids/Species in April were higher than those in September in all treatments. In the Temperature+4 °C treatment, the germination peaks in April were higher than those in September, and the differences varied from 10 to 72%, where P. Shan-tong had the smallest and *P. tomentosa*, together with *P. fortunei*, had the greatest. In the Temperature+20 °C treatment, the germination peaks in April were higher than those in September, and the differences varied from 30 to 55%, where P. Shan-tong had the smallest and *P. fortunei* had the greatest. In the Temperature−15 °C treatment, the germination peaks in April were higher (except for those of *P. tomentosa* and *P. fortunei*, which had higher germination peaks in September than in April) than those in September, and the differences varied from 14 to 45%, where *P. elongata* had the smallest and P. Shan-dong had the greatest. Moreover, the germination peaks of Hybrids/Species were the highest in the Temperature+4 °C treatment. The second highest peaks appeared in the Temperature+20 °C treatment (on average 27% and 29% lower than those in the Temperature+4 °C treatment in April and September, respectively). The lowest peaks appeared in the Temperature−15 °C treatment (those in the Temperature+4 °C treatment in April and September were, on average, higher by 267% and 180%, respectively, and those in the Temperature+20 °C treatment in April and September were higher by 179% and 116%, respectively). Species (i.e., *P. elongata*, *P. tomentosa* and *P. fortunei*) had higher peaks in almost all cases than all Hybrids in the Temperature+4 °C and Temperature+20 °C treatments in April and September; however, in the Temperature−15 °C treatment, Species had lower peaks than all Hybrids.

### 2.4. Mean Daily Germination

**Figure 4:**
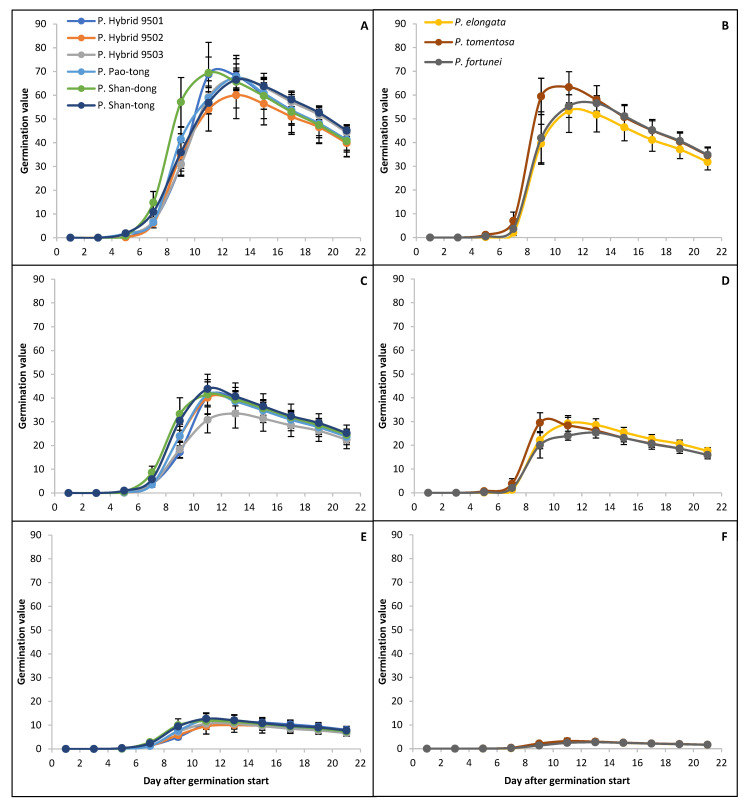
Germination values of *Paulownia* in April. (**A**,**B**)—the Temperature+4 °C treatment; (**C,D**)—the Temperature+20 °C treatment; (**E**,**F**)—the Temperature−15 °C treatment. Whiskers denote standard deviations.

**Figure 5:**
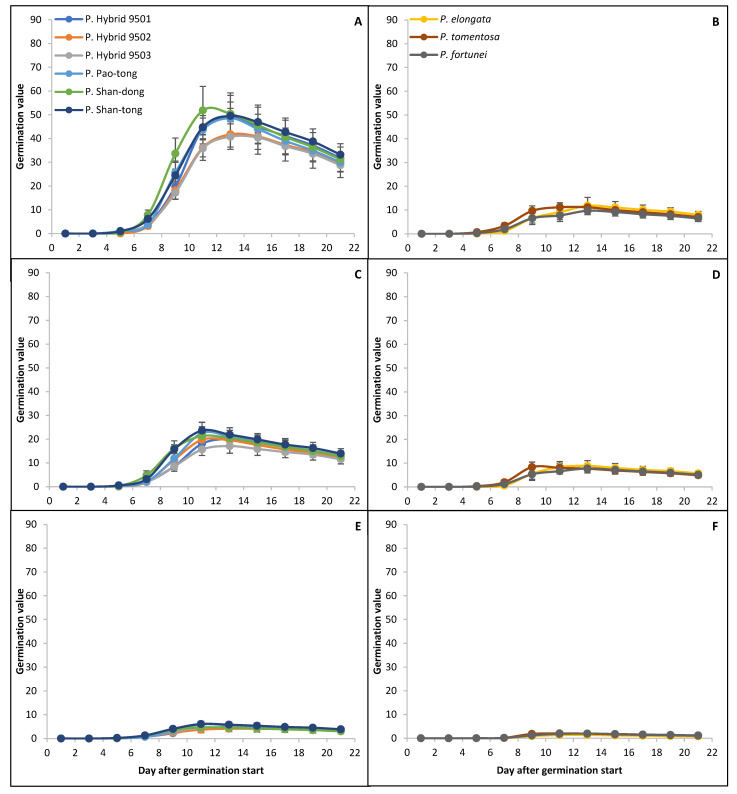
Germination values of *Paulownia* in April. (**A**,**B**)—the Temperature+4 °C treatment; (**C**,**D**)—the Temperature+20 °C treatment; (**E**,**F**)—the Temperature−15 °C treatment. Whiskers denote standard deviations.

**Figure 6:**
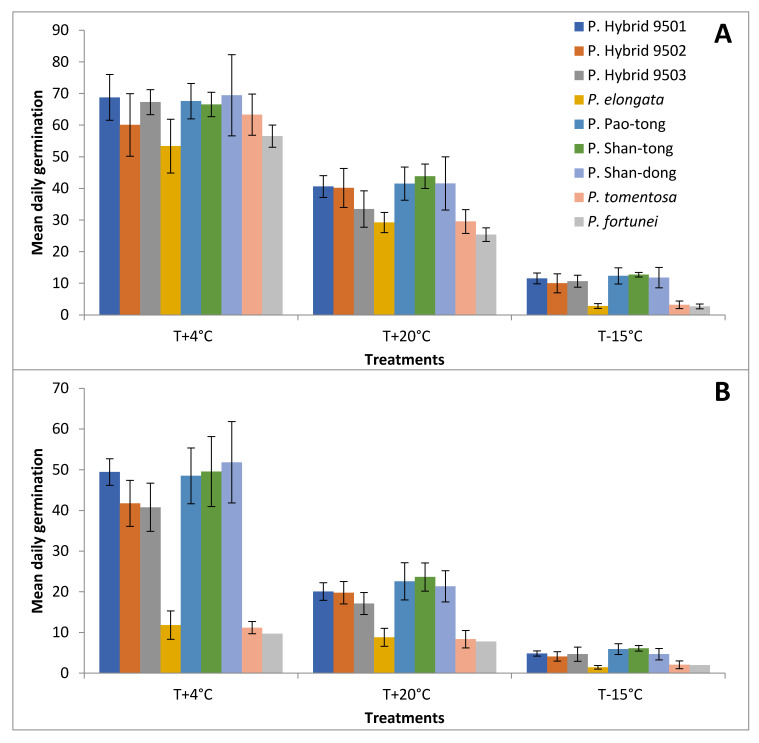
Mean daily germination of *Paulownia* in April (**A**) and September (**B**). T+4 °C—the Temperature+4 °C treatment; T+20 °C—the Temperature+20 °C treatment; T−15 °C—the Temperature−15 °C treatment. Whiskers denote standard deviations.

The seeds of all *Paulownia* Species/Hybrids germinated in the first week of the experiment in both April and September (Figure 4 and Figure 5). In April, the germination values (GV) increased quickly, and we recorded the peaks (Figure 6) of all Species/Hybrids from the middle to the end of the second week in all treatments. P. Hybrid 9501, P. Hybrid 9503, P. Pao-tong, P. Shan-dong, and P. Shan-tong had the highest peaks—almost 70 in the Temperature+4 °C treatment in April. In this treatment, P. Hybrid 9502 and *P. tomentosa* had the second highest peaks and *P. elongata* and *P. fortunei* the lowest (53 and 56, respectively). Statistically significant differences occurred only between the highest and lowest peaks (*p* values were in the range of 0.0148–0.0367). In the Temperature+20 °C and Temperature−15 °C treatments in April, the group with the highest peaks comprised all Hybrids and the group with the lowest comprised all Species (*p* values between Hybrids and Species were in the range of 0.0001–0.0312). From the peaks, the GVs of all Hybrids and Species in all treatments slowly decreased.

In September, the differences between Hybrids and Species were visible in all sections of the curves (i.e., the increase, the peaks and decrease) in all treatments. While Hybrids always increased very quickly and, after reaching high peaks, decreased slowly, Species increased slowly, reached only low peaks, and then decreased very slowly. Statistical differences between the peaks of Hybrids and Species were always 0.0001. In the Temperature+4 °C treatment, Hybrids were split into two statistical groups, according to the heights of their peaks. Group 1 consisted of P. Hybrid 9501, P. Pao-tong, P. Shan-dong, and P. Shan-tong, and group 2 of P. Hybrid 9502 and P. Hybrid 9503 (*p* values were in the range of 0.0186–0.0455). There were no statistical differences among Hybrids in the Temperature+20 °C and Temperature−15 °C treatments.

## 3. Discussion

In April, the germination rate of the seeds was 70% (Hybrids) and 55% (Species) when they were stored at +20 °C, and in September it was around 50% (Hybrids) and 35% (Species). These germination rates are relatively low and do not correspond with the observations of Liu et al. [46], according to whom almost all seeds should germinate. Also, Barnhill et al. [47] observed a high germination rate of seeds left at room temperature, and Bonner & Burton [48] showed a high germination rate, 86%, after nine days.

After cold stratification, the germination rates were more than when the seeds were stored at +20 °C: 90% (Hybrids) and 60% (Species) when the seeds germinated in April and around 80% (Hybrids) and 40% (Species) in September. However, after cold stratification, almost 100%, 90%, or 85% of all seeds should germinate, according to Carpenter & Smith, Liu et al., or Bonner & Karrfalt [13,46,49], respectively.

It seems that the germination rate can be increased via cold stratification. Therefore, we can deduce that *Paulownia* is dormant. However, Toda & Isikawa, Borthwick et al., and Pasiecznik [50,51,52] state that *P. tomentosa* seeds do not enter dormancy or that it is very shallow. On the other hand, according to Liu et al. [46], *P. elongata* shows non-deep dormancy. Kuppinger, Bonner & Karrfalt, and Oung & Young [10,49,53] distinguish seed dormancy relative to the time that the seeds fell from the tree. *Paulownia* seed is not dormant immediately after falling from its capsule during late summer and the beginning of autumn [11]. It becomes dormant (whether still on the tree or fallen) when exposed to unfavorable conditions (for a brief period at lower temperatures) or is soaked with water in the dark [54,55]. This secondary dormancy can change germination time and germination speed, and it can reduce or eliminate the need for light or the temperature range, within which germination comes about; and if some mechanism does not disturb dormancy, the seeds do not germinate [10,47,56,57]. In the past, smoke, high-exposure to light, exposure to mineral soil, or adequate moisture were used for awakening from dormancy [47,51,57,58]. In the present, cold stratification of different durations (i.e., around 3–4 °C; the duration of action varies, according to many authors), pre-soaking, red light (not far-red light), exposure to exogenous GA_3_, etc., are used in the laboratory [46,59,60,61].

The germination rate, energy, and the other parameters were always higher for seeds that had germinated in April than for those in September. All the seeds used in this study were produced in the previous growing season. They should therefore have the highest germination capacity. This is confirmed by many authors [13,62,63] who found that germination is highest during the first two years, and by Bonner & Karrfalt [49], who stated that germination is at its highest within the first three years after seed production. However, there are differences in germination among individual seasons. Seeds can be sown in all seasons, where the most common and appropriate for most plants is spring [64]. Winter temperatures help to overcome dormancy of seeds sown in autumn and winter, and they germinate in spring [65,66] or after two years [67], depending on their life cycles. Wahlenberg [68] recorded that seeds of western white pine sown in the spring show more germination and germination energy than in autumn.

*Paulownia* comes from China [2], and it grows in areas where the northern border is located on ca the −5 °C isotherm and the temperature at the highest altitude is around −10 °C [2]. *Paulownia* is mostly frost-hardy, for example, *P. tomentosa* tolerates temperatures down to −22 °C, *P. elongata* and *P. catalpifolia* in the range of −15 °C to −18 °C, and *P. fortunei* and *P. kawakamii* in the range of −5 °C to −10 °C. These values were measured for plants and not seeds. *Paulownia* seeds are orthodox [69], which means that they can endure extreme frost [70]. Seeds of many orthodox species are generally frost-tolerant down to −196 °C [71]; however, the typical temperature used is above −20 °C [72]. Unfortunately, they lose frost hardiness with the onset of germination [73]. Baskin & Baskin [69] state that germinating seeds can either grow or wither. Seeds of *P. tomentosa*, *P. elongata*, and *P. fortunei* had very low values of all parameters both in the spring and in the autumn when we put them into the refrigerator with the temperature set at −15 °C. We expected similar values because Hyatt [74] observed that *Paulownia* seeds rarely, if at all, germinate in wildlands in southeastern Pennsylvania, where the climate is humid continental with a warm summer [75]. We recorded a germination rate of up to 17% with Species. On the other hand, 15% of seeds germinated in natural conditions in Ohio [15], which is also located in a humid continental climate [75]. This value is similar to our Species values. However, the seeds of Hybrids achieved even higher values (almost 30%), which is alarming. According to many authors who found the spreading of *Paulownia* via seeds [2,10,11,12,13,14,15,16,17,18,19,20], it is obvious that these species can have the potential to become invasive, which our results confirm; however, our findings show that seeds of *Paulownia* hybrids have greater potential than those of artificially uncrossed species.

## 4. Materials and Methods

### 4.1. Plant Material

Small plantings or very large plantations are usually established with seeds obtained from common sellers or using plant material from forest nurseries that also purchase seeds. For this experiment, all seeds (that had been collected in the fall of 2021) were purchased from three different sellers to ensure that the material used was diverse. Nine Species/Hybrids were bought; each seller had more than 9 species and hybrids of *Paulownia*, but only the ten listed below could be purchased from all three. Moreover, the sellers had to declare that the seeds were collected in the fall of 2021. The acquired seeds were stored in marked permeable bags under controlled conditions (i.e., a constant temperature of +10 °C, low air moisture, and away from direct sunlight and fluorescent lighting [76]), which does not break dormancy up to beginning of the preparatory part of the first experiment.

#### 4.1.1. Species

*Paulownia tomentosa* (“*P. tomentosa*”; synonym: *Bignonia tomentosa*, *Incarvillea tomentosa*, *Paulownia grandifolia*, *P. imperialis*, *P. lilacina*, *P. recurva*) occurs at an altitude of up to 1800 m a.s.l. [9] with precipitation from 500 to 1500 mm and a temperature range from −20 °C to +40 °C [2].

*Paulownia elongata* (“*P. elongata*”) is one of the fastest-growing species of *Paulownia*, producing a lot of biomass [77,78]. It occurs at altitudes of up to 2000 m a.s.l. [9] with precipitation from 600 to 1500 mm and a temperature range from −15 °C to +40 °C [2].

*Paulownia fortunei* (“P. fortunei”; synonym: *P. duclouxii*, *P. meridionalis*, *P. mikado*) plays important roles in wood production, farmland, and environmental protection [79], and is susceptible to phytoplasma infection during its growth and development, resulting in a large number of clumps of indefinite buds, thereby reducing the yield and quality of *Paulownia* wood substantially [2]. It occurs at altitudes of up to 1100 m a.s.l. with precipitation from 1200 to 2500 mm and a temperature range from −10 °C to +40 °C [2].

#### 4.1.2. Hybrids

Paulownia Hybrid 9501 (“P. Hybrid 9501”; synonym: P. Royal Treeme; P. Nordmax 21) is a hybrid of *P. fortunei* and *P. tomentosa* and is a fast-growing plant resistant to disease, cold, frost and drought [80]. It can be a source of quality saw timber, but the branches grow to greater diameters with unsuitable silvicultural treatments, which devalues quality of saw timber [5].

Paulownia Hybrid 9502 (“P. Hybrid 9502”) is a hybrid of *P. fortunei* and *P. tomentosa*, which grows fast, produces high-quality wood and is resistant to disease, frost and drought [5].

Paulownia Hybrid 9503 (“P. Hybrid 9503”) is a hybrid of *P. fortunei* and *P. tomentosa*, which grows fast, produces high-quality wood and is resistant to disease, frost and drought and, moreover, it has fine branches [5].

Paulownia Pao-tong Z07 (“P. Pao-tong”) is a hybrid of *P. fortunei*, *P. tomentosa* and *P. kawakami* [81] with the greatest recorded frost resistance (down to temperatures as low as −33 °C). It is a fast-growing plant with a cylindrical stem and is therefore suitable for saw timber [5].

Paulownia Shan Dong (“P. Shan Dong”) is resistant down to −25 °C and is suitable for extreme climate conditions [82]. It is used in the form of wood chips in the production of furniture [5].

Paulownia Shan-tong (“P. Shang-tong”) is a hybrid of *P. fortunei* and *P. tomentosa* and is used in the form of biomass and as saw timber. It is highly resistant to drought and frost [5].

### 4.2. Experimental Design

*Paulownia tomentosa* is the most widespread species of *Paulownia* and is often marked as an invasive or casual species, unlike other *Paulownia* species, which is why it was used as a reference in this study.

The twenty-one-day experiment was initiated twice:

- On 20 April 2022, the experiment imitated the natural germination of seeds in the spring;
- On 29 August 2022, the experiment was repeated with worse conditions before the beginning of the experiment (i.e., longer storage of seeds).

In the preparatory part (i.e., three months before the beginning) of the experiment on 20th April, the seeds were pulled out from the controlled conditions. Half of the seeds were used for the experiment on 20th April 2022, and the second half (in their bags) was spread in controlled conditions (i.e., a constant temperature of +20 °C, air moisture of 50% and away from direct sunlight and fluorescent lighting).

In the preparatory parts of the two experiments, we started preparations. For each individual Species/Hybrid, we set up 18 units of 100 (purchased) seeds each—six units of seeds from each of the three sellers. The seeds of each unit were spread out on a separate filter paper inside a marked germinator. The two units (i.e., 2 germinators) from the first seller, the two from the second, and the two from the third—6 units in total—were used for one treatment. The following three treatments were performed:

- Temperature+20 °C: Fifty-four germinators (eighteen from each seller), where six always contained the same individual Species/Hybrid, were laid out on germination tables at a temperature of +20 °C and air moisture of 60–70%. Seed moisturizing was performed before the start of the experiment. This temperature was chosen as the maximum temperature for storing seeds, and, moreover, these seeds were not exposed to lower temperatures.
- Temperature+4 °C: Fifty-four germinators (eighteen from each seller), where six always contained the same individual Species/Hybrid, were opened, moisturized, closed, and placed inside a refrigerator (with a constant temperature of +4 °C and air moisture of 80%). Each week, the filter papers were observed and moisturized whenever necessary. This temperature was chosen as the temperature necessary for the breaking of dormancy naturally.
- Temperature−15 °C: Fifty-four germinators (eighteen from each seller), where six always contained the same individual Species/Hybrid, were opened, moisturized, closed, and placed inside a refrigerator (with a constant temperature of +4 °C and air moisture of 80%). Each week, the filter papers were observed and moisturized whenever necessary. In the middle of the period, the units were placed inside a freezer (with a constant temperature of −15 °C and air moisture of 40%) for two weeks and, subsequently, returned into the refrigerator. This temperature was chosen as one of the lowest temperatures (in winter months) that most areas located in Dfa and Dfb (i.e., Central and Eastern Europe and 40–50° North latitude of North America) reach [75].

After three months, the germinators that had been left on the germination tables were opened, moisturized, and closed. All other units placed inside the refrigerator were taken out and placed on germination tables. All units were observed every 2 days for a period of 21 days, during which the germinated seeds were counted and removed from the test. A seed was considered germinated upon the appearance of the primary leaf.

Data was processed and evaluated, with focus on the following parameters:

- The dynamic of germination: A curve representing the dynamic of germination during the first 21 days after setting up the germination trial.
- The germination rate (%): The number of germinated seeds at the end of the experiment (i.e., after 21 days) out of the total number of seeds [83].
  (1) $$GR=\frac{N_{g}}{N_{t}},$$
  where
  GR—germination rate;
  N_ge_—number of germinated seeds;
  N_t_—total number of seeds.
- The germination energy (%): The number of germinated seeds (recorded until the peak) divided by the total number of seeds [84].
  (2) $$GE=\frac{N_{g}}{N_{t}},$$
  where
  GE—germination energy;
  N_ge_—number of germinated seeds;
  N_t_—total number of seeds.
- The mean daily germination (MDG): The highest value of all germination values (GV; a vigor index, which combines total percentage of seed germination and speed to evaluate seed lot quality) [83,85,86]. Each GV is calculated in three steps for each observation. Step 1: Calculation of the daily germination speed (DGS; i.e., cumulative count of germinated seeds divided by the number of days from the start of the experiment) for each observation. Step 2: The sum of the DGSs divided by the number of observations (the seeds were observed each second day, i.e., the number of observations was always half the number of days). Step 3: The DGS multiplied by the cumulative count of the germinated seeds divided by 100 and multiplied by 10.
  (3) $$DGS=\frac{N_{g}}{N_{day}}$$
  where
  DGS—daily germination speed;
  N_g_—number of germinated seeds;
  N_day_—number of days from the start of the experiment to observation.
  (4) $$GV=\frac{\sum DGS}{N_{observation}}\times \frac{\sum N_{g}}{100}\times 10$$
  where
  GV—the highest value of all germination values;
  DGS—daily germination speed;
  N_observation_—number of observations;
  N_g_—number of germinated seeds;
  N_day_—number of days from the start of the experiment to observation.

### 4.3. Statistical Analysis

Data in this study was processed in Microsoft^®^ Excel 2010 (Microsoft Corporation, Redmont, WA, USA). Statistical analysis of the data was performed using TIBCO Statistica™ 14.0.0 (TIBCO Software Inc., San Ramon, CA, USA) with a confidence interval of 95%. Normality of the data distribution was examined by the Shapiro–Wilk test before the main analysis. The main effects (i.e., Species/Hybrids and treatments) were analyzed using two-way analysis of variance (ANOVA), after which Fisher’s LSD test was applied to identify differences among the Species/Hybrids and treatments and their statistically homogenous groups.

### 4.4. Limitations

We bought the seeds from sellers. Although these sellers declared that the seeds were collected in fall 2021, they may have been older and their storage may not have been in accordance with the relevant standards. The results (especially in the case of the +4 °C treatment) indicate that the conditions were within acceptable limits. Another very important factor influencing this study is the number of samples. This study required a great number of germinators and much space on the germination tables, and therefore we set up only 6 samples (of 100 seeds each) from each Species/Hybrid per individual treatment: 162 germinators in April and 162 in August in total. The 16,200 seeds (with a gradually decreasing number) on the germination tables were monitored every other day, and each germinated seed had to be removed. Furthermore, we tried to reduce human error to a minimum, and the authors always worked in pairs. Despite the possibility of minor errors that were made during the experiment and the smaller number of samples, the results should be homogeneous and relevant.

## 5. Conclusions

We asked two basic questions about germination of *Paulownia*: “Does *Paulownia* have varying seed germination among species and hybrids?” and “Can they become invasive via their seeds?” We performed an experiment in which we split the seeds into three groups, each of which was left at one of the following temperatures:

(1) +20 °C throughout the entire experiment;

(2) +4 °C three months before the start of the experiment;

(3) +4 °C three months before the start of the experiment and at −15 °C for two weeks in the middle of the +4 °C phase.

Subsequently, we determined their germination. The results are alarming. The values of the germination rate and energy of *P. tomentosa*, *P. elongata* and *P. fortunei* were very high in the Temperature+20 °C and Temperature+4 °C treatments because it was thanks to these temperatures that the dormancy was broken. The values were lower in the Temperature−15 °C treatment; however, the germination rate stayed at around 20%. On the other hand, our tested hybrids had a germination rate of almost 100% in the Temperature+4 °C treatment and slightly less (more than 70%) in the Temperature+20 °C treatment. It is important to add that the values of the germination rate and the other parameters in the Temperature−15 °C treatment were double those of Species. Based on our results and on those of other authors, it is possible to say that not only *P. tomentosa*, but also *P. fortunei* and *P. elongata* seem to be invasive. However, hybrids of *Paulownia* have an even greater potential to be invasive.

## Data Availability

The data is available from the first author upon request.
